# Household clusters reveal household- and variant-specific properties of SARS-CoV-2

**DOI:** 10.1017/S0950268821002600

**Published:** 2022-01-07

**Authors:** Udo Buchholz, Kai Schulze-Wundling, Matthias an der Heiden

**Affiliations:** Department for Infectious Diseases, Robert Koch Institute, Berlin, Germany

**Keywords:** Household cluster, variant, cumulative case number, interval to secondary cases

## Abstract

It is unclear if – after symptom onset of a primary case of coronavirus disease-2019 (COVID-19) in a household – ensuing chains of transmissions among household members occur and if household epidemiology of COVID-19 is modified by the different circulating variants. We analysed data of 52 774 household clusters to investigate the day of symptom onset of ensuing cases in households relative to the symptom onset of the primary case within the household. Irrespective of cluster size or age of the primary case, 95% of all secondary household cases had symptom onset within 14 days after the symptom onset of the primary case. Stratification by variant showed that the mean interval from symptom onset of the primary case to the symptom onset of secondary cases decreased significantly from 4.8 days (wildtype) to 4.5 days (alpha) and 4.0 days (delta). Similarly, the cumulative proportion of 95% of secondary cases occurred within 14 days (wild type), 12 days (alpha) and 10 days (delta). Our findings suggest that during dominant delta circulation – apart from rare individual constellations – a 10-day household quarantine after symptom onset of the primary case is sufficient for household contacts who remain COVID-free.

## Introduction

Many governments and public health authorities attempt to control the coronavirus disease-2019 (COVID-19) pandemic by means of isolating symptomatic cases as well as testing, tracing and quarantining their close contacts [1, 2]. Quarantine of close contacts is defined as the isolation of healthy persons who had a high-risk exposure to a confirmed COVID-19 case. It is unclear if and how often chains of transmissions occur among households with more than two persons which would justify prolonged quarantine of uninfected household contacts. Thus, based on observations of the infectiousness and incubation period, 10 days of isolation after symptom onset for COVID-19 cases and 14 days of quarantine for close contact persons after the last potentially infectious contact or exposure to a confirmed case have been recommended by the WHO, ECDC, as well as many national public health agencies (such as the Robert Koch-Institute in Germany) [2–4]. For uninfected household contact persons, this amounts to at least 24 days (10 days infectiousness of primary case + 14 days quarantine) of quarantine.

To date, little empirical data about the temporal transmission dynamics in households of different sizes and a different number of COVID-19 cases exist [5, 6]. Also, it is unclear if household epidemiology differs between variants, such as wild type, Alpha and Delta-variant. To improve evidence-based quarantine recommendations for households we analysed German surveillance data of reported household clusters.

## Methods

In Germany, laboratory-confirmed cases of COVID-19 must be reported to one of 380 local public health authorities (LPHA) who investigate each case and send the information to the state health departments who again transmit case data after verification to the Robert Koch-Institute. This is a legal requirement of the mandatory reporting system specified in the German ‘Infektionsschutzgesetz’ (‘Protection against Infection Act). The data are partially available for the public, but not in the detail necessary to undertake the following analyses.

In general, asymptomatic contact persons were not tested, but contact persons were followed up regarding symptoms through daily monitoring, and were tested for severe acute respiratory syndrome coronavirus 2 (SARS-CoV-2). When at least two cases are identified as belonging to the same household they are grouped as household clusters. Household size is not reported. We analysed data of household clusters with information on symptom onset of all cases involved. We included cases that were transmitted to RKI from the beginning of the pandemic until 24 September 2021.

First, we defined the primary household cases as those with the first symptom onset. In particular, for some outbreaks, two or more cases had the onset on the same day. If another household member had an identical date of symptom onset, the primary case was assigned randomly. We investigated (1) the distribution of symptom onsets after the day of symptom onset of the primary case, stratified by the number of secondary cases in the household clusters and (2) the distribution of symptom onsets stratified by the age of the primary case.

Secondly, to investigate potential differences between the three variants ‘wildtype’, Alpha and Delta, we analysed three different time periods when the respective variant dominated. We attributed infections from calendar week (CW) 10 until 48/2020 to the wild type, from CW 12-22/2021 to Alpha and from CW 27-38/2021 to Delta. To compare the difference of the mean interval of secondary cases' symptom onset after symptom onset of primary cases statistically we used the Kruskal–Wallis test. Analysis was performed with Stata (Stata Corporation 2021, Stata Statistical Software: Release 17. College Station, TX: StataCorp LLC).

## Results

145 933 cases in 52 774 household clusters with the latest date of symptom onset of the primary case on 24 September 2021 were eligible for inclusion. Clusters with more than six cases (*n* = 681 clusters with 5610 cases) were excluded because it was unclear whether household outbreaks involving more than six cases were still individual households. Of 52 093 remaining clusters with 140 323 cases, 29 483 (57%) were clusters of two cases, 12 997 (25%) of three cases, 6500 (12,5%) of four cases, 2312 (4%) of five cases and 801 (1,5%) were clusters of six cases. On average the outbreaks contained 2.7 cases. Household outbreaks with children (less than 18 years old) tended to be a little larger, they contained on average three cases.

Cases were a median of 38 years old, 50% of cases were between 21 and 53 years old (interquartile range; Table 1). Both sexes were represented almost equally (51% female, 49% male).

**Table 1.** Age groups, numbers, proportion and cumulative proportion of all cases and only primary cases included in the analysis (*N* = 140 323^a^); Germany, 2021

	All cases			Primary cases		
Age group	Number of cases	%	Cumulative %	Number of cases	%	Cumulative %
0–9	11 502	7	8	3793	6	6
10–19	19 622	13	22	6962	11	17
20–29	21 379	15	37	10 152	15	32
30–39	21 729	15	53	11 352	17	49
40–49	22 688	16	69	11 563	18	67
50–59	23 436	18	86	11 837	18	84
60–69	11 725	9	94	6150	9	94
70–79	5416	4	98	2856	4	98
80–89	2510	2	99.8	1248	2	99.8
90+	297	0.2	100	117	0.2	100
Total	140 304	100	100	66 030	100	100

a For 19 cases age is unknown.

**Fig. 1.**
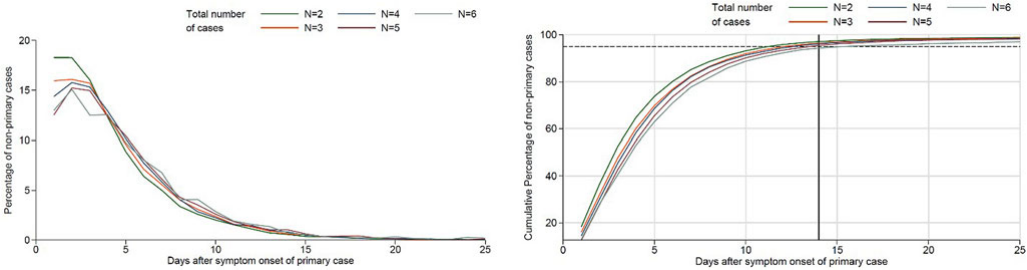
Second, 3rd, 4th, 5th and 6th cases in household clusters of COVID-19 by day of symptom onset counted after symptom onset of the primary case (day 0). Left panel: number of cases by day of symptom onset after symptom onset of the primary case. Right panel: cumulative proportion; dashed horizontal line at 95%, vertical line at 14 days.

Our analyses show that the proportion of secondary cases starts high and peaks within the first 5 days of the symptom onset of the primary case (Fig. 1, left panel). The proportion of secondary cases declines afterwards steadily until approximately 2 weeks after the onset of the primary case. Irrespective of the size of the household cluster, all cases have occurred in about 95% of cases until the 14th day after the onset of symptoms of the primary case (Fig. 1, right panel). There is no indication of waves of transmission within households.

In addition, we investigated the distribution by age of the primary case. 0–9-year-old children were primary cases in 6%, 10–19-year-old cases in 11% and adults between 20 and 59-year-old between 15% and 18% (Table 1). Both the distribution by day of symptom onset and the cumulative proportion of household cases with symptom onset until the 14th day after symptom onset of the primary case are quite independent of the age of the primary case (Fig. S1). In the 10-year age groups, the cumulative proportion is 95% or higher in all age groups.

**Fig. 2.**
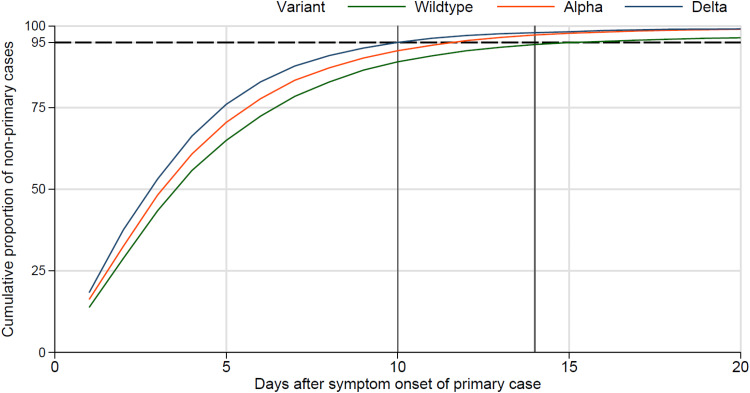
Cumulative proportion of secondary household cases stratified by SARS-CoV-2 variant; dashed horizontal line at 95%, vertical line at 10 and 14 days.

Stratification by the SARS-CoV-2 variant suggests a decreasing time to onset of secondary household cases from wildtype to Alpha and Delta (Fig. 2). The curves of the cumulative proportion of secondary household cases ‘shift’ to the left, indicating that a larger proportion of secondary cases have their symptom onset within a shorter interval after symptom onset of the primary case. For example, during the period in which the wildtype variant was dominant, 95% of secondary cases had their symptom onset within 14 days after the symptom onset of the primary case. However, during the period in which Delta was the dominant variant, 95% of secondary cases had their symptom onset within only 10 days of the primary case. Accordingly, the mean interval from symptom onset of the primary case to the symptom onset of secondary cases decreased significantly from Wildtype (4,80; 95% confidence interval (CI) 4,75–4,85), to Alpha (4,50; 95% CI 4,46–4,54) and Delta variant (4,02; 95% CI 3,95–4,09).

## Discussion

Our analysis of over 50 000 German household clusters revealed three key empirical findings.

First, the proportion of cluster cases after symptom onset of the primary case was highest between days 1 and 5 after the onset of the primary case and not at day 5 or later. We assume that there are two reasons for this. Intense exposure of household contacts likely occurs already before symptom onset of the primary case (presymptomatic exposure), resulting in many secondary cases with a symptom onset shortly after symptom onset of the primary case. Secondly, in some households there may have been not only one primary case (even though their day of symptom onset differed), but two or even more co-primary cases instead, for example when two persons of a household became infected at the same event (outside of the household). We are unable to identify these households based on data alone, but they may pull the average of the interval between symptom onset of the primary case and secondary cases towards the primary case. Therefore, we refrained from calling the interval between symptom onset of the primary case and symptom onset of secondary cases the ‘serial interval’ although in many cases it probably is.

The second main finding was that almost all secondary cases occurred until the 14th day after symptom onset of the assumed primary case, irrespective of the cluster size or age of the primary case. There is little data in the literature for comparison, but an Israeli study reported that household outbreaks ‘typically last(ed) 2–4 weeks’ [5]. However, households in that study were frequently very large (mean 5.3 household members). Another study reported that 86% of secondary cases had their infection within 10 days [6]. The findings of our study concur with these findings and extend the conclusions to a broader spectrum of household (cluster) sizes, reflecting the entire age spectrum. As we have seen no indication of waves of transmission among households with more than two cases, this suggests that additional cases do not increase the overall risk of household members to become infected, or said differently, the risk of a household member to become infected is largest at the beginning of the exposure conferred by the primary case. The highest risk may in fact exist during his or her presymptomatic (or early symptomatic) phase, when the primary case may often not be aware of the infection and will not be isolating himself or herself, but viral shedding and transmission risk is already substantial [7–9].

Thirdly, stratified analyses by variant revealed a continuous shortening of the interval between symptom onset of secondary household cases and symptom onset of the primary case. This finding is in line with recent virologic evidence suggesting faster viral replication, greater infectiousness and higher viral load of Delta during the early period of infectiousness [10, 11]. This implies that persons infected with Delta reach the infectious shedding dose earlier (i.e. the latency period has shortened), leading to a shorter serial interval even without a longer incubation period. A higher viral load, in turn, may lead to a higher infectious inoculation dose of exposed persons, which could lead to faster development of infection and a shorter incubation time. We cannot differentiate between the two explanations with our data. Our finding of the increasingly shorter interval from primary to the secondary case moving from the wildtype to the Alpha and then Delta variant may be the result of a shorter serial interval, which could be the result of a shorter latency, a shorter incubation period, or a combination of both.

Our study is subject to the following limitations: (1) data are not derived from a study setting but from mandatorily reported data. However, the database is large, and it is reassuring that even without study conditions findings seem quite robust. In our view, it shows the potential of using routinely collected surveillance data to formulate policy recommendations. (2) Because only those households with information on symptom onset of all household members were included, our findings do not directly apply to contexts with asymptomatic cases. It is possible that, for example, teenagers in the household are more likely to be asymptomatic and are more likely to be infected later because of more distant contact behaviour. Nevertheless, our findings should be generalisable to asymptomatic household cases as well.

Supported by our results, Germany reduced in September 2021 the period of a 14-day quarantine for uninfected household members to 10 days. In particular, there is no need for ‘chain quarantine’, i.e. that a quarantine of yet uninfected household members starts from zero when a second household member becomes infected. The rule was kept that any household contact experiencing symptoms compatible with COVID-19 should be tested and additional laboratory-confirmed cases occurring in a household need to be isolated for 10 days after her or his own symptom onset. Although there is a residual risk from the 5% of cases that may have an onset post-quarantine which could be quite significant given high community transmission quarantine rules are subject to a trade-off between ‘strictness’ and compliance that needs to be considered – the longer and more complex the quarantine rules are (i.e. ‘chain-quarantine’ of 24 days) the less compliance could be expected. In addition, household members released from quarantine are asked to minimise the number of contact persons until day 20 after symptom onset of the primary case and, if ill, should seek health care to become tested.

In summary, the mean interval of symptom onset of secondary cases after symptom onset of the primary case decreased significantly over time, suggesting an increasingly shorter serial interval. No transmission waves within households were observed. Lastly, during the Delta phase, the cumulative proportion of 95% of secondary cases occurred within 10 days after symptom onset of the primary case justifying a 10-day quarantine for household contact persons.

## Data Availability

The research presented in this paper is based on data that was routinely collected by LPHA and reported the Robert Koch-Institute in accordance to §4 of the German Protection against Infection Act (IfSG, BGBl. I S. 1045). Due to privacy regulations, the data is securely stored at Robert Koch-Institute and cannot be made available to the public by law.

## Supplementary material

For supplementary material accompanying this paper visit https://doi.org/10.1017/S0950268821002600. click here to view supplementary material
