# Supplementary material for: Household clusters reveal household- and variant-specific properties of SARS-CoV-2
Source: Epidemiol Infect. 2022 Jan 7;150:e17. doi: 10.1017/S0950268821002600 (PMC8770840; doi:10.1017/S0950268821002600)

**Supplementary Figures**

**Figure S1**

Secondary household cases in relation to the age group of the primary case. Left panel: number of cases by day of symptom onset after symptom onset of the primary case. Right panel: cumulative proportion; dashed horizontal line at 95%, vertical line at 14 days.


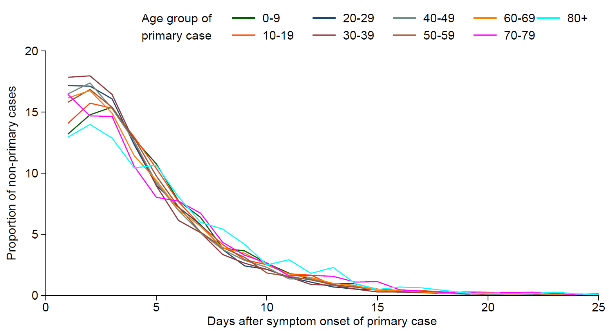

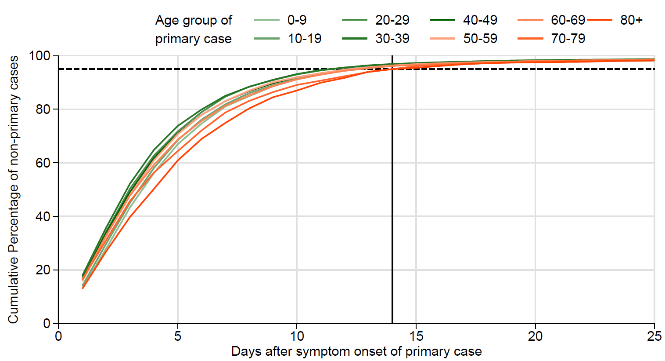

Supplement: Supplementary file 1 [file S0950268821002600sup001.docx]
